# Using RNA-Seq to Explore the Repair Mechanism of the Three Methods and Three-Acupoint Technique on DRGs in Sciatic Nerve Injured Rats

**DOI:** 10.1155/2020/7531409

**Published:** 2020-06-07

**Authors:** Tao-tao Lv, Yan-jun Mo, Tian-yuan Yu, Shuai Shao, Meng-qian Lu, Yu-ting Luo, Yi Shen, Yu-mo Zhang, Wong Steven

**Affiliations:** School of Acupuncture, Moxibustion and Tuina, Beijing University of Chinese Medicine, Beijing, China

## Abstract

**Objective:**

To study the effects of the three methods and three-acupoint technique on DRG gene expression in SNI model rats and to elucidate the molecular mechanism of the three methods and three-acupoint technique on promoting recovery in peripheral nerve injury.

**Methods:**

27 male SD rats were randomly divided into three groups: a Sham group, the SNI group, and the Tuina group. The Tuina group was treated with a tuina manipulation simulator to simulate massage on points, controlling for both quality and quantity. Point-pressing, plucking, and kneading methods were administered quantitatively at Yinmen (BL37), Chengshan (BL57), and Yanglingquan (GB34) points on the affected side once a day, beginning 7 days after modeling. Intervention was applied once a day for 10 days, then 1 day of rest, followed by 10 more days of intervention, totally equaling 20 times of intervention. The effect of the three methods and three-point technique on the recovery of injured rats was evaluated using behavior analysis. RNA sequencing (RNA-Seq) analysis of differentially expressed genes in DRGs of the three groups of rats was also performed. GO and KEGG enrichment was analyzed and verified using real-time PCR.

**Results:**

RNA-Seq combined with database information showed that the number of differentially expressed genes in DRG was the largest in the Tuina group compared with the SNI group, totaling 226. GO function is enriched in the positive regulation of cell processes, ion binding, protein binding, neuron, response to pressure, response to metal ions, neuron projection, and other biological processes. GO function is also enriched in the Wnt, IL-17, and MAPK signaling pathways in the KEGG database. PCR results were consistent with those of RNA sequencing, suggesting that the results of transcriptome sequencing were reliable.

**Conclusion:**

The three methods and three-acupoint technique can promote the recovery of SNI model rats by altering the gene sequence in DRGs.

## 1. Introduction

Peripheral nerve injury (PNI) refers to the peripheral nerve plexus, nerve trunk, or its branches injured by external forces (such as crush injury, traction injury, and contusion). The repair and regeneration of PNI and the recovery of its related functions are of primary clinical importance and have always been a popular research focus. Injured axons of the peripheral nervous system have the ability to regenerate successfully [1], and repair after injury is related to functional recovery. Pathological stimulation or damage to the peripheral nervous system can lead to neuropathic pain [2] such as ectopic pain, burning sensation, hyperalgesia, and spontaneous or induced unpleasant sensory abnormalities, which are widely believed to be caused by overexcitation of primary sensory neurons and ectopic activation of voltage-gated ion channels, including sensory neuron-specific voltage-gated sodium channel 1.8 (NaV1.8) [3]. Axonal injury causes muscle weakness and sensory loss [4]. The prevalence of neuropathic pain is as high as 8.0%, which not only causes physical pain but also increases the incidence of depression, anxiety, and other emotional disorders, greatly affecting the quality of life of patients [5]. Previous studies over the past decade have proved that the three methods and three-acupoint technique can improve the behavioral indices of pain and temperature, promote the expression of NTFs, protect neurons, repair nerve myelin sheathes, and prevent muscle atrophy in rats with peripheral nerve injury over the past decade [6–8]. But what gene expression changes do the three methods and three-acupoint technique intervention induce in SNI rats and what processes have they promoted that aid in the repair of peripheral nerve injury? The involvement of biological processes and transduction pathways is not clear. In this study, RNA-seq technology was used to reveal the relevant transcriptome changes and conduct in-depth and thorough analysis, which is conducive to the development and maintenance mechanisms of peripheral nerve injury and provides a theoretical basis for massage treatment of peripheral nerve injury.

The dorsal root ganglion (DRG) receives all nerve impulses sent from peripheral receptors, including general somatosensory and visceral sensations. DRG axons bifurcate into two independent branches: the peripheral branches dominate the skin and muscles and the central branch enter the spinal cord from the DRG and terminates at the surface of the dorsal horn, which then transfers sensory information to the central nervous system. After peripheral nerve injury and axon degeneration, neurotrophic factor secretion changes in DRG neuron structure occur involving molecular and metabolic processes from a variety of cells and are involved in multiple signaling pathways, accompanied by a large number of changes in gene expression [9].

In degree II-III nerve injury, the injury response includes the response of the injury site, Wallerian degeneration (WD) below the level of injury, the response of the nerve cell body above the level of injury, and the response of the peripheral organs. The Sciatic nerve crush model is a specific regeneration model occurring after peripheral nerve injury [10–13]. In this study, the sciatic nerve injury (SNI) model was used to stimulate the degree II-III injury of the peripheral nerve by clamping the nerve axon [14]. In the early stage of the injury, the axon and myelin sheath were broken. Ischemia occurred in the injured nerve segment, and intranervous edema was obvious. WD caused granular decomposition of the structure of the distal axon, followed by irregular swelling of the myelin sheath, structural destruction, and rapid disintegration. Neuroplasty can restore the signal balance of the nerve pathways and provide various nutritional factors for nerve growth and regeneration by correcting the changes of the microenvironment and repairing various abnormal and damaged nerve structures that lead to pain.

Through the use of surgery, hormones, nerve growth factors, and other drugs, nerve function can generally only recover about 70% and allogenic stem cell transplantation for the treatment of sciatic nerve injury is not particularly effective [14]. Opioids can effectively alleviate symptoms, but long-term use of opioids will produce hyperalgesia and tolerance, thus reducing the effectiveness [15]. In the SNI model, the injured sciatic nerve is close to the central nervous system, far from the effector, and the nerve has not yet recovered. The function of the distal effector, especially the muscle, has been lost. Therefore, it is of great significance to make the nerve recover early through peripheral intervention. Tuina is a manual manipulation of traditional Chinese medicine for central nervous system disorders and functions as a way of peripheral intervention. Tuina refers to the practitioner's manipulation of a mechanical force with the appropriate stimulus on the body surface or deep tissue of the subject, causing changes in the receptors in the local skin or deep tissue. The stimulus from mechanical force is then converted into electrical signals and passed through the afferent fibers in the form of nerve impulses. According to Chinese medicine, tuina manipulation can dredge channels and collaterals, promote qi and blood circulation, dispel cold, and relieve pain [16]. It can also effectively improve the swelling and degeneration of primary nociceptive neurons in DRGs of SNI rats. Apoptosis and dissolution of neurons in the DRGs after light nerve injury can protect the morphological integrity of primary sensory neurons in the sensory pathway.

RNA-sequencing (RNA-Seq) technology is a high-throughput sequencing technology which has developed rapidly in recent years. It can study gene function and gene structure as a whole and reveal the molecular mechanism of specific biological processes and disease occurrence. RNA-Seq technology is relatively low in cost, has good repeatability, and can be detected with only a small sample size, especially for the analysis of biological samples that are difficult to obtain [17]. RNA-Seq analysis of DRG transcriptome changes after nerve injury is helpful to reveal the mechanism of peripheral nerve injury and may lead to finding new targets for the prevention and treatment of neuropathic pain [18]. RNA-Seq analysis of DRG in rats with crush injury of the sciatic nerve showed that the maximum permeability of nerve membranes appeared within 4–7 days after crush injury. This timing corresponded to the peak of acute inflammation [19]. The overall inflammatory response at the site of nerve injury was increased on the 7th day after injury. Therefore, this study used the rat sciatic nerve clamp injury model to simulate clinical peripheral nerve injury. After 7 days of injury, the three methods and three-acupoint technique were used to treat the nerve-injured rats [1]. RNA-Seq technology was used to study gene expression changes at the level of DRG transcription and explore the repair mechanism of the three methods and three-acupoint technique on nerve-injured rats, so as to provide genetic evidence supporting tuina treatment of peripheral nerve injury.

## 2. Materials and Methods

### 2.1. Animals

Twenty-seven male Sprague-Dawley (SD) rats of 6-7 weeks' weight (200 ± 10 g) were fed in the laboratory of Beijing University of Chinese Medicine (SPF level) and provided with food and water freely. All rats were provided by Sparford Biotechnology Co., Ltd. The feeding temperature was 23 ± 2°C, the humidity was 45%, and the light and dark period was 12 hours (turning on the light at 8 a.m.). Adaptive feeding occurred for 1 week. The Committee on Animal Protection and Use of Beijing University of Chinese Medicine (BUCM-3-20151202-4001) approved all procedures used in this study to minimize the number of animals used and the suffering of animals. Rats were randomly divided into a Sham group (*n* = 9), the SNI group (*n* = 9), and the Tuina group (*n* = 9).

### 2.2. Modeling Methods

Fasting and water deprivation occurred 24 hours before modeling. Rats were anaesthetized by intraperitoneal injection of 1% pentobarbital sodium (350 mg/kg body weight).

In the SNI group and the Tuina group, rats were fixed in the prone position and the sciatic nerve was found and clamped for 5 seconds at 5 mm distal to the sciatic nerve nodule with fine toothless vascular forceps, with a full buckle (5 kg by pressure test), resulting in injury points about 4 mm in length. We then administered local saline irrigation, layer by layer disinfection, suturing, and disinfection. The Sham group rats only were exposed to surgery where the sciatic nerve was found but not manipulated, and the other steps were the same as the SNI group.

The rats were kept warm, and we awaited the awakening of rats. All of the rats fasted for 24 hours postoperatively, but were allowed free access to water. The wound condition and foot changes were observed regularly after operation.

### 2.3. Intervention Methods

The sham group and SNI group were fed routinely, and they were held in the restraint for 9 minutes every day, without tuina intervention.

The Tuina group was administered the three methods and three-acupoint technique as the intervention for the SNI model rats. The treatment began on the 7th day after the model was established. The “Massage and tuina manipulation simulator” (patent No. ZL 2007 0187403.1, smooth spherical surface with a diameter of 10 mm) was used daily to stimulate Yinmen (BL37), Chengshan (BL57) and Yanglingquan (GB34) points on the affected side of the rats using three methods: point-pressing method, plucking method, and kneading method. The stimulating force was 4 N, and the stimulating frequency was 60 times per minute; each method and each point were used for 1 minute, totaling 9 minutes (1 min/acupoint *∗* 3 methods *∗* 3 acupoints) [20]. Treatment was administered once a day for 10 days, with 1 day of rest, and then 10 more treatments for a total of 20 times. In order to reduce the stress response of animals, petting and catching of the animals were administered for 9 minutes every day before the formal intervention.

### 2.4. Behavioral Measurement

The accumulated pain score was used to assess pain intensity [21]. We observed the landing and weight-bearing situations of the two hind paws (the hind paws are compressed and whitened to indicate weight-bearing): if the hind paws do not touch the ground, a scoring of 2 points was assessed; if the hind paws do not bear weight, it was scored as 1 point; and if the hind paws land and bear weight, a scoring of 0 points was assessed. The assessment occurred every 5 minutes and lasted 1 minute long; the most commonly used posture within the 1 minute was assessed as the standard; 12 assessments were conducted for a total of 1 hour observation. The cumulative pain score was obtained by subtracting the contralateral foot score from the affected foot score. Each rat was measured three times, and the average value was obtained.

### 2.5. RNA Extraction and Construction of Sequencing Library

DRG of L4-L6 was taken from the right side of each group after 20 treatments. In order to obtain enough RNA, the DRGs of every three mice were aggregated to extract RNA as a sample, and three biological replicates (*n* = 9 mice/group) were performed in each group. The total RNA was extracted from tissue samples using the “Guanidine isothiocyanate-phenol-chloroform extraction” method, and the concentration and purity of RNA were detected by Nanodrop2000. The integrity of the RNA was assessed by agarose gel electrophoresis, and the RNA integrity number (RIN) was determined by Agilent2100. The total RNA content was 1 *μ*g, the concentration was more than 50 ng/uL, and the OD260/280 was between 1.8 and 2.2. Samples with a RIN score >8 were used for sequencing.

In this project, transcriptome sequencing was completed based on Hiseq sequencing platform, and the illumine PE library was constructed by Illumina truseqtm RNA sample prep Kit Method for 2 × 150 bp sequencing. QuantiFluor® dsDNASystem was used to quantify and mix the data in proportion. Clusters were generated by bridge PCR amplification on cBot. The quality control of the sequencing data was carried out, and then the transcriptome data were analyzed by bioinformatics. Fragments per kilobase per million mapped reads (FPKM) value was used to determine gene expression level, and differential gene expression was analyzed with the full transcriptomic data of each sample.

### 2.6. GO and KEGG Analyses

Gene ontology (GO) terms are used to describe and classify the functions of genes and proteins. Goatools software was used for GO enrichment analysis of gene concentration, and the Fisher exact test was used. When the corrected *P* value (FDR) <0.05, it is considered that the GO function is significantly enriched.

Kyoto Encyclopedia of Genes and Genomes (KEGG) is a large knowledge base for systematic analysis of gene functions and association of genomic information and functional information. Enrichment analysis used the KEGG pathway as a unit. Statistical tests were used to calculate the FDR (q-value) of the *P* value and the *P* value corresponding to each pathway. Corrected *P* Value (0.05) was used as a threshold to identify which pathway was significantly enriched in differentially expressed genes compared with the whole genome background.

### 2.7. Real-Time PCR Analysis

The primers listed were amplified (Table 1). The reverse-transcription reaction conditions were 95°C for 3 min × 1 cycle, 95°C for 30 s, 60°C for 30 s, and 72°C for 40 s × 35 cycles. The main mixtures were prepared and the cycle conditions were set (predenaturation at 95 for 2 min, denaturation at 95 for 15 sec, annealing at 63 for 30 sec, and elongation at 72 for 30 sec). The 96-well plate of the sample was placed in the Bori LineGene9600plus fluorescence quantitative PCR system of reaction.

### 2.8. Statistical Analysis

The data were analyzed by ANOVA to test the uniformity of variance, and then the *t* test was carried out in SPSS22.0. The significant level of statistical analysis was defined as *P* < 0.05.

## 3. Results

### 3.1. Behavioral Results

There was no redness and swelling in the operation area, the wound healed well, and the health condition was good after operation. One day after modeling, the hind limbs of the rats in the Sham group moved freely. The knee joints of the rats in the SNI group and Tuina group were straight and unable to flex and the foot drooped and they displayed limping on the operative side, which indicated the success of the SNI model preparation. On the 7th day after modeling (intervention day 0), the rats in the Sham group were able to move their hind limbs freely, while the rats in the model group and the Tuina group had a slight toe valgus, limp gait, and suspended foot or were unable to land. On the 28th day after modeling (after 20 interventions), the hind limbs of the rats in the Sham group moved freely; the hind limbs of the rats in the SNI group continued to suffer from claw contracture, the soles of hind feet were completely suspended and could not touch the ground, the joints were stiff, and the hind limb muscles were clearly atrophied. The hind limbs of rats in the Tuina group could bear weight, and the feet could be laid flat on the ground. The toes could be placed on the ground, they could be separated, and walking with a limb was possible.

### 3.2. Comparisons of Cumulative Pain Scores in Rats

**Figure 1:**
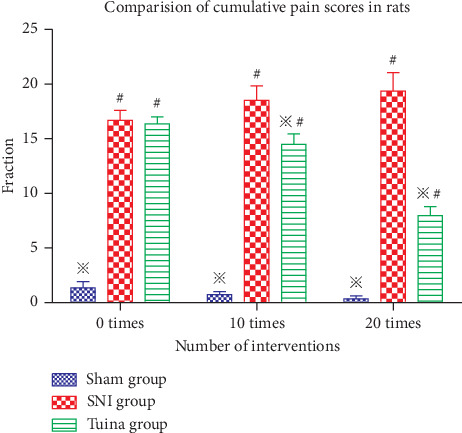
Comparisons of cumulative pain scores of rats in each group. ^※^Compared with the SNI group, *P* < 0.05; ^#^compared with the Sham group, *P* < 0.05.

Before the establishment of the model, the rats in each group landed on the ground with two hind claws and were loaded with weight. The cumulative pain score of each group was the same, all of which were 0. The results showed that (Figure 1) after 7 days of massage intervention, the cumulative pain score of the SNI group and Tuina group was significantly higher than that of the Sham group (*P* < 0.05); after 10 times and 20 times of intervention, the cumulative pain score of the Tuina group was significantly better than that of the SNI group (*P* < 0.05). However, there was still a certain difference between Sham group and Tuina group (*P* < 0.05), suggesting that the three methods and three-acupoint technique can alleviate the pain sensation of rats with nerve injury to a certain extent.

## 4. Bioinformatics Analysis of DRG in Rats

### 4.1. Analysis Results of Quality Control

In order to ensure the quality of the original sequencing data, it is necessary to evaluate the quality of the original data before analysis. The results showed that 430 million reads were obtained, with an average of about 47.76 million reads per sample; *Q*20 of all samples ranged from 97.97% to 98.36%, *Q*30 from 94.01% to 94.97%, and 95.93% to 96.92% of all samples could map to the reference genome, indicating that the sequencing results were reliable (Table 2).

### 4.2. DEG Analysis Results

**Figure 2:**
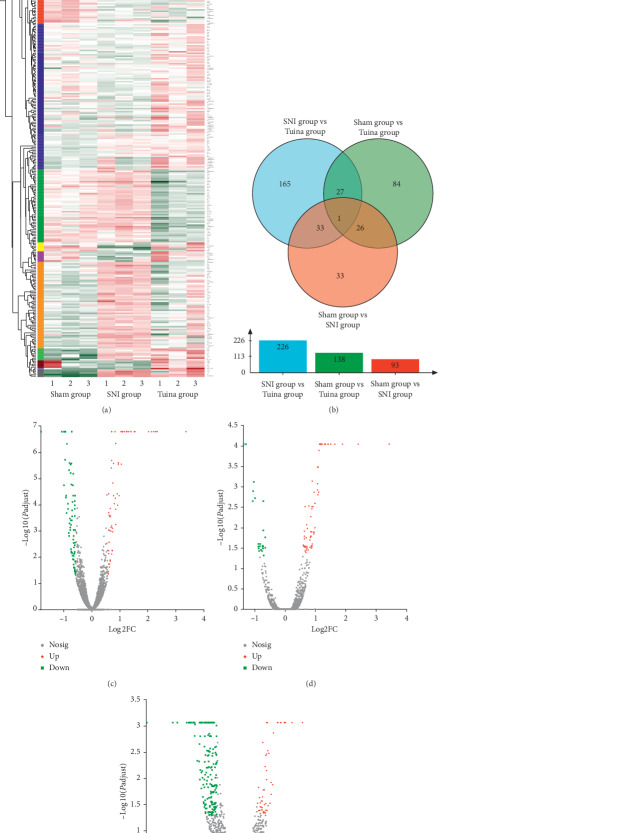
Analysis of differentially expressed genes. (a) Each column in the graph represents a sample, and each row represents a gene. The color in the graph represents the size of the gene expression in the sample. Red represents the higher expression of the gene in the sample, while green represents the lower expression. See the number label under the upper left color bar for the change trend of the specific expression. On the left is the tree graph of gene clustering and the module graph of subclustering, on the right is the name of the gene. The closer the two gene branches are, the closer their expression is. On the top is the tree graph of sample clustering, and on the bottom is the name of sample. (b) Genes, in which the circles of different colors in the numerical generation represent the common or unique number of genes between two or three sets of gene sets, the sum of all numbers in the circle represents the total number of genes in the gene set, and the cross region of the circle represents the common number of genes among the gene sets. (c), (d), and (e) volcanic maps are used to represent two groups of DEGs. Horizontal and vertical axes show the difference of gene expression in different groups. The smaller the *P* value, the more significant and the larger the difference—log 10 (adjusted *P* value). Different genes have different splashes. There is no significant difference between grey dot genes, red dot genes, and green dot genes.

After 20 interventions, the DRG genome of rats in each group was sequenced using RNA. There were 369 DEGs in the three groups. Three groups of DEG two-dimensional hierarchical cluster analysis can clearly show degree of separation between the Sham group, the SNI group, and the Tuina group (Figure 2(a)). A Venn diagram showed that there were 226 differentially expressed genes between the Tuina group and SNI group (Figure 2(b)). Volcanic map analysis showed that the distribution of DEGs in DRG of each group had statistical significance. The differential gene expression of DRG in rats with peripheral nerve injury and the three methods and three-acupoint technique was determined. There were 93 DEGs in the Sham group compared with the SNI group, 23 genes were upregulated (Figure 2(c)), and 70 genes were downregulated in the Sham group compared with the Tuina group. There were 138 DEGs in Sham group compared with Tuina group, of which 66 genes were upregulated and 72 genes were downregulated (Figure 2(d)); 226 DEGs were differentially expressed in the Tuina group and SNI group, of which 50 genes were upregulated and 176 genes were downregulated (Figure 2(e)). There are many differentially expressed genes in DRG under the intervention of the three methods and three-acupoint technique. Combined with the behavioral changes of the rats, it is speculated that the change of pain perception of SNI rats treated with the three methods and three-acupoint technique is due to the change of DRG genes.

### 4.3. Analysis Results of GO and KEGG Analysis

**Figure 3:**
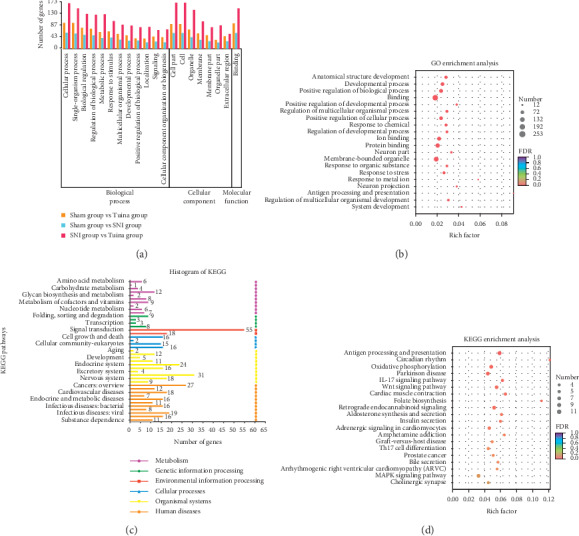
Analysis results of GO and KEGG. (a) The ordinates in the graph represent the secondary classification terms of GO, the abscissas represent the number of genes in the secondary classification, and the colors represent different gene sets. (b) The vertical axis represents the GO term, and the horizontal axis represents the ratio of rich factor to the number of annotated genes. The larger the rich factor is, the greater the degree of enrichment is. The size of the dot represents the number of genes in this GO term. The color of the dot corresponds to different FDR (Pvaule_corrected) ranges. (c) The ordinate is the name of the KEGG metabolic pathway; the abscissa is the number of genes or transcripts annotated to the pathway. (d) The vertical axis represents the pathway name, and the horizontal axis represents the ratio of rich factors to the number of annotated genes. The larger the rich factor, the greater the degree of enrichment. The size of the dot indicates the number of genes in the pathway, and the color of the dot corresponds to different *Q* value ranges.

In order to describe the function of DEGs in DRG of SNI rats after peripheral nerve injury and after intervention with the three methods and three-acupoint technique, the GO enrichment analysis of DEGs in each sample was carried out based on the GO database. The results show that the top 20 GO secondary classification terms include biological regulation processes, response to stimuli, and positive regulation of biological processes (Figure 3(a)). In addition, there are biological functions such as presynaptic processes, movement, growth, and molecular sensor activity involved in chemical synaptic transmission. GO function is enriched in biological processes such as active regulation of the development process, positive regulation of cell processes, ion binding, protein binding, neurons, membrane-bound organelles, response to organic substances, response to pressure, response to metal ions, neuron projection, and so on (Figure 3(b)).

DEGs were screened by R-script with gene expression changes greater than 1.5 times and *P* < 0.05. Pathway significance analysis was performed. A total of 251 KEGG annotations were found. Pathway classification statistics showed that the metabolic classification included energy metabolism, degradation, and metabolism of exogenous organisms, nucleotide metabolism, lipid metabolism, accessory factors, and vitamin metabolism, and genetic information processing included folding, classification, degradation, and translation. Including signal transduction and signal molecule interaction, in the biological system, the top three are the immune system (31), endocrine system (24), and nervous system (18); human diseases include cancer, immune diseases, endocrine metabolic diseases, and neurodegenerative diseases; cell processes include cytogenesis length and apoptosis, transport, and catabolism (Figure 3(c)). A total of 244 enrichment pathways were identified by KEGG enrichment analysis.  Figure 3(d) shows that KEGG ranks the top 20 important pathways, including Wnt signaling pathway, IL-17 signaling pathway, MAPK signaling pathway, oxidative phosphorylation, cholinergic synapses, and HIF-1 pathway. There are 5 metabolic pathways with significant differences (*P* < 0.05).

## 5. Results of Real-Time PCR

**Figure 4:**
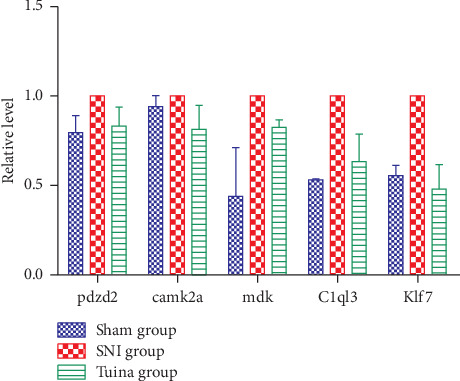
RT-PCR expression results. The abscissa is the gene name, and the ordinate is the relative expression.

In order to verify the transcriptome sequencing results and further analyze the expression of genes that play an important role in repairing injured nerves after massage intervention, five representative genes, Pdzd2 (ENSRNOG00000013140), and Camk2a (ENSRNOG00000018712), were selected in this study. Real-time PCR validation was performed on MDK (ENSRNOG00000017560), C1ql3 (ENSRNOG00000017459), and Klf7 (ENSRNOG00046242). The results showed that, compared with the Sham group, the expression of Pdzd2, Camk2a, mdk, C1ql3, and Klf7 (genes involved in neuronal synaptic regulation) increased in the SNI group, while the expression of these genes decreased in injured rats after three-point intervention. The expression trend of these genes was consistent with that obtained by RNA-Seq, indicating that RNA sequencing data could reliably reflect changes in gene expression. KEGG data show that the Wnt signaling pathway, which Camk2a participates in, is closely related to the MAPK signaling pathway, which is consistent with the analysis of the pathway in this study. These results suggest that the effects of the three methods and three-acupoint technique on nerve repair and regeneration in SNI model rats may be carried out by these genes (Figure 4).

## 6. Discussion

In this study, we identified changes in DRG at the transcriptome level after peripheral nerve injury and we further explored the changes of DRG transcriptome after peripheral intervention. Peripheral intervention can significantly inhibit the exacerbation of peripheral nerve injury, promote the regeneration and repair of the injured nerve, gradually reduce inflammatory edema in the injured part of the sciatica, accelerate the removal of degeneration, necrosis and disintegration products, improve local microcirculation, improve the recovery rate of the sciatic nerve function index, and shorten the period of injury of the sciatic nerve. After recovery, the abnormal rate of nerve conduction velocity and latency was improved. As a peripheral intervention method, the clinical efficacy of Chinese tuina in the treatment of peripheral nerve injury has been established [22–24], but the relevant mechanism is not clear enough. At present, many studies have focused on specific genes that change after nerve injury, but there is a lack of research on gene repair of injured tissues by therapeutic methods, which has been neglected in previous studies on RNA-seq and peripheral nerve injury.

In this study, SD rats were used as experimental subjects, and the SNI model was used to simulate clinical peripheral nerve crush injury. The three methods and three-acupoint technique were selected as the intervention method. RNA-Seq technology was used to quantitatively estimate the gene expression level of FPKM and to explore the changes of DRG gene sequence using the three methods and three-acupoint technique in rats with nerve injury. In “three methods,” the point-pressing method has the functions of warming meridians and activating collaterals, regulating qi flow, and is mostly used for pain relief. The plucking method has the functions of relieving spasm, separating adhesion, treating numbness, and pain caused by nerve compression. The kneading method is an important method to relieve muscle spasm and also can relieve pain of injured areas [25]. The “three points” are Yinmen Point (BL37), Chengshan Point (BL57), and Yanglingquan Point (GB34); the “three points” are located on the Foot-Taiyang Bladder Meridian and Foot-Shaoyang Gallbladder Meridian, which conform to the principle of selecting points along meridians and local points in acupuncture and moxibustion; the “three points” are located on the sciatic nerve and its branches; Yinmen Point is located on the sciatic trunk and Chengshan Point on the tibial nerve branches of the sciatic nerve. The upper and Yanglingquan acupoints are located on the common peroneal nerve, a branch of the sciatic nerve, and the three acupoints are surrounded by the biceps femoris, gastrocnemius, and anterior tibial muscle, respectively. Therefore, manipulation as these “three points” can stimulate acupoints, nerves and muscles at the same time, so the three stimulating sites are called “acupoint-nerve-muscle areas.”

GO analysis confirmed that differentially expressed genes in DRGs participate in many biological processes, including biological regulation, response to stimuli, presynaptic processes involved in chemical synaptic transmission, motility, and molecular sensor activity. They were also enriched in ion binding, neuron, membrane-bound organelles, and pressure. Biological processes include reaction to metal ions and neuron projection. These results show that the three methods and three-acupoint technique can promote the repair of peripheral nerve injury through many potential mechanisms. This experiment focused on the recovery of axons of injured nerves using the three methods and three-acupoint technique. The results showed that the three methods and three-acupoint technique could change DRG gene expression, promote axon growth and pain recovery of injured nerves, and ultimately improve peripheral nerve injury in SNI rats. This is consistent with the previous research of our group. The KEGG pathway is enriched in the Wnt signaling pathway, IL-17 signaling pathway, MAPK signaling pathway, oxidative phosphorylation, cholinergic synapse, and so on, which indicates that the changes at the gene level in the three-method three-point technique repair of the injured nerve pathway are related. Previous studies have shown that Wnt signaling is an important pathway for neuropathic pain [26, 27]; IL-17 is a potential therapeutic target for neuropathic pain, which contributes to the neuroinflammatory response and hypersensitivity to pain after neuropathic injury [28], and p38 MAPK plays an important physiological role in nerve regeneration [29]. It is important to control the occurrence of inflammation in the recovery from nerve damage. Wnt signaling pathway and IL-17 signaling pathway represent the main categories. In this study, more than seven genes of the Wnt signaling pathway were expressed differently after nerve injury. RT-PCR verified Camk2a, an important gene of the Wnt signaling pathway, and demonstrated the importance of the Wnt signaling pathway in the repair of peripheral nerve injury, which is related to previous nerve injury. The research of concerning gene expression in wound repair is consistent.

Peripheral nerve regeneration is a complex process. Initial peripheral axon injury will prompt sensory neurons to prepare for subsequent regeneration after peripheral or central axon injury, which activates the intrinsic regeneration ability of DRG neurons and is regulated by a variety of molecular reactions and signaling pathways [30]. The RNA-Seq data of this study showed that the intervention of the three methods and three-acupoint techniques could reduce injury to the peripheral nerve and increase the expression of Pdzd2, Camk2a, mdk, C1ql3, and Klf7 genes after injury. The protein interaction module of the PDZ domain plays an important role in the interaction between synaptic junction components [31]. The synaptic junction accumulates various molecules involved in neurotransmission and synaptic plasticity. Voltage-gated sodium channel NaV1.8 is only expressed in nociceptive neurons and plays an important role in the pain pathway. Results show that Pdzd2 and P11 play a synergistic role in regulating the expression of NaV1.8 in sensory neurons [32]. GO analysis showed that BP function of Camk2a was related to neuronal synaptic plasticity regulation, MAPK signaling cascade, Wnt signaling pathway, calcium regulation pathway, and dendritic morphogenesis. CC function included postsynaptic density and presynaptic membrane. Mice lacking Camk2a expression in neurons had spatial learning, memory deficit, and fine motor. Behavioral changes include impaired skills, subtle changes in social interaction, and decreased dendritic spine density [33]. The RNA-Seq data in this study showed that Camk2a was upregulated after peripheral nerve injury and downregulated during the process of three-method and three-point intervention repair. This indicates that Camk2a is a key gene for the treatment of peripheral nerve injury using massage. Midkine (Mdk) is a growth factor involved in the development and repair of various tissues, especially nerve tissue. The lack of Mdk after peripheral nerve injury is a key factor for degeneration and regeneration. It acts as a repair neurotrophic factor. Manipulating the supply of Mdk can provide an interesting therapeutic option for the treatment of peripheral nerve injury [34]. This study shows that the BP function of Mdk involves the negative regulation of neuronal apoptosis. C1q13 is a secreted neuronal protein, and previous studies have shown that C1q13-deficient mice exhibit fewer excitatory synapses and different behavioral abnormalities [35]. GO analysis showed that the biological function of C1q13 BP includes the regulation of synaptic tissue. RNA-Seq showed that the gene expression of C1q13 in DRG of rats changed significantly before and after nerve repair. Klf7 is a transcription factor that stimulates the proliferation and axon regeneration of Schwann cells (SC) after peripheral nerve injury. It is a promising therapeutic transcription factor in nerve injury [36]. Studies has observed an increase of CTB-labeled neurons in DRG, filament, and P0 and S100 in Hargreaves test and further supported KLF7 in the injured nervous system, where it acts as a growth-promoting transcription factor [37]. Combined with the results of RNA-Seq and RT-PCR, we found that the regulation of Klf7 gene expression may be a potential therapeutic strategy for peripheral nerve injury.

In summary, promoting peripheral nerve injury recovery using the three methods and three-acupoint technique involves a variety of GO biological functions and changes of gene expression in multiple KEGG pathways and promotes the repair and regeneration of injured nerves through multiple physiological processes. Differential gene expression analysis using RNA-seq not only provides information about a selected group of genes but also provides information about the entire mouse transcriptional profile. These genes may have unrecognized functions, because only a few of the first 20 up- and downregulated genes have been scientifically proven to have neurorepair-related functions. The primary disadvantage of this study is that there is only a single time point for data collection. Future research will combine different time points to collect and analyze data in order to study dynamic biological processes more extensively and better explain the mechanism of repairing pain dysfunction of injured nerves. Furthermore, we will further study the functions of differentially expressed genes that have not yet been identified, so as to promote our understanding of the mechanism of nerve repair and to prevent and treat peripheral nerve injury.

## 7. Conclusion

**Figure 5:**
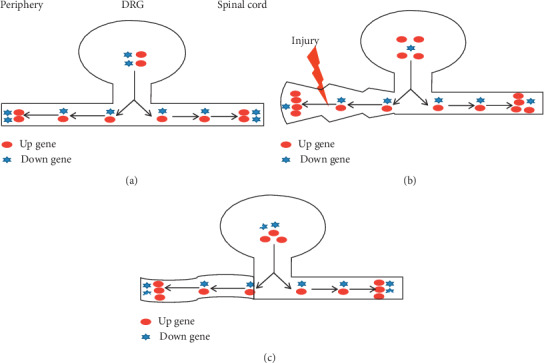
Diagram of repair mechanism of DRG in rats with nerve injury intervened by three methods and three acupoints. (a) Sham group. (b) SNI group. (c) Tuina group.

After peripheral nerve injury, the expression of some genes in DRG increases or decreases. The three methods and three-acupoint technique acting on SNI rats can reduce the expression of upregulated genes and increase the expression of downregulated genes in the DRG, so that the expression of genes tends towards normal and therefore can promote the repair and regeneration of peripheral nerve injury (Figure 5). The RNA-seq data obtained in this study have been proven to be repeatable and verified by RT-PCR. This study presents the theoretical support for clinical massage treatment of peripheral nerve injury and provides a scientific basis for its prevention and treatment of neuropathic pain.

## Figures and Tables

**Table 1:** RT-q PCR primers.

Gene	Primer name	Sequence (5′--3′)
1	pdzd2-F	CCGCAGAGAAGGAACTTCAG
	pdzd2-R	CTTCCCTGGGGAACAGAGAT

2	camk2a-F	TCTTGTTAGCCTGGCCTAGC
	camk2a-R	CCAGAAGGGAAGGAACTGAC

3	mdk-F	ACTGGAAGAAGGAGTTTGGA
	mdk-R	TTGTACCGAGCCTTCTTCAG

4	C1ql3-F	CCAGCTCTTCCACTGTTAGG
	C1ql3-R	AGTGCTGGTTCTCATAAGGC

5	Klf7-F	ACAGCTACACAGCCGTCAAC
	Klf7-R	GCCACCAGTTTCAACGTCAC

Actin	F18	CACCCGCGAGTACAACCTTC
	R224	CCCATACCCACCATCACACC

**Table 2:** Quality control and sequencing information for samples.

	Sample	Raw reads	Clean reads	*Q*20 (%)	*Q*30 (%)	Total mapped
1	Sham_1	45425774	44558476	98.08	94.29	42984668 (96.47%)
2	Sham_2	45461770	44827548	98.36	94.97	43406830 (96.83%)
3	Sham_3	50811396	50173584	98.24	94.66	48599893 (96.86%)
4	SNI_1	46687088	45980852	97.97	94.01	44426768 (96.62%)
5	SNI_2	43868392	43103046	98.17	94.53	41348676 (95.93%)
6	SNI_3	47101016	46211164	98.2	94.6	44530159 (96.36%)
7	Tuina_1	52310780	51732204	98.34	94.93	50068003 (96.78%)
8	Tuina_2	50010464	49467140	98.3	94.82	47945406 (96.92%)
9	Tuina_3	49023202	48449138	98.23	94.64	46953793 (96.91%)

(1) Sample: sample name, 9 cDNA libraries are Sham group (Sham_1, 2 and3), SNI group (SNI_ 1, 2 and 3), Tuina group (Tuina _1, 2 and 3); (2) Raw reads: counting the number of original sequence data; (3) Clean reads: counting the number of sequencing data after filtering; (4) *Q*20, *Q*30: counting Phred values, respectively. Total mapped: the number of clean reads that can be located on the genome.

## Data Availability

The raw data we obtained from RNA-Seq are available in the National Center for Biotechnology Information (NCBI) Sequence Read Archive (SRA) with submission number SUB6659208.
